# Health and safety practices and policies concerning human exposure to RF/microwave radiation

**DOI:** 10.3389/fpubh.2025.1619781

**Published:** 2025-07-21

**Authors:** James C. Lin

**Affiliations:** Department of Electrical and Computer Engineering, University of Illinois Chicago, Chicago, IL, United States

**Keywords:** cellular mobile telecommunication, assumptions for the standards, military industrial complex, industry-regulatory complex, WHO-EMF systematic reviews, ICNIRP and WHO-EMF, paradigm shift

## Abstract

Concerns about the impacts on the public health and safety of radiofrequency (RF) exposure are increasing with the rapid proliferation of cellular mobile telecommunication systems and devices. There is also lack of confidence surrounding the applicability of stated health safety rules, limits and guidelines for RF exposure including their use for 5G and the expected 6G. This paper: (1) considers the currently promulgated standards for safe human exposure to RF radiation, (2) examines assumptions underlying the standards, (3) describes the roles of the military industrial complex in influencing research on the health effects and standards setting for safety levels, (4) discusses the engagement of an industry-regulatory complex, (5) explains the interaction between ICNIRP and the WHO-EMF, (6) scrutinizes recent publications of WHO-EMF commissioned systematic reviews, and (7) concludes with some observations on an apparent paradigm shift.

## Introduction

In 2021, there were nearly 15 billion cellular mobile devices operating worldwide. The number of RF mobile devices is expected to reach 18 billion by 2025, an increase of 4 billion devices compared to 2020 levels (1). The current world population is about 8.2 billion according to the most recent United Nations estimates (2). These numbers suggest that currently each person alive on Earth is subjected to the exposure of two or more RF radiating devices. Likewise, about 97% of adults in the U.S. own a mobile phone or cellular device including 99% of those between the ages of 18–29 and 94% of the population residing in rural areas (3).

The fast spread of cellular and mobile telecommunication devices and systems is raising concerns about the health impacts and safety of radiofrequency (RF) exposure. There is also unease about the effectiveness of publicized health safety rules, limits, and standards for RF radiation used by these devices and systems.

President D. Eisenhower delivered his farewell address, on January 17, 1961, which has since been regarded as one of the most famous speeches in American history. It warned Americans, among other stuff about the “military-industrial complex,” a web of individuals and institutions involved in the development of military hardware and production of weapons. Eisenhower warned that the “United States must guard against the attainment of unwarranted influence by the military-industrial complex,” which included the Department of Defense, members of Congress, privately and corporately owned military industries and contractors. Eisenhower believed that “the military-industrial complex tended to promote policies that might not be in the country’s best interest.”

The military-industrial complex played a huge role in influencing research on the biological effects from exposure to electromagnetic fields and waves including RF radiation since the inception of related scientific investigation in the middle of the 20th century (4–7). It was strongly directed toward tissue heating induced by RF radiation to elevate the body temperature. Ever since, the military industrial complex has tended to be highly critical of research studies that suggested otherwise and defended rigorously the status quo (8).

This paper critically assesses the issues of biological effects and health implications that are applicable to cellphone and mobile telecommunication uses of RF and microwave radiation. It begins with a historical perspective and assesses the currently promulgated standards for safe human exposure. It examines the assumptions underlying the standards. It also discusses (1) the degree in which the military-industrial complex was involved in promoting research on the biological effects and setting of safety standards; (2) the seeming engagement of an industry-regulatory complex; (3) important laboratory results that the guidelines do not take into consideration; (4) the interaction between ICNIRP and the WHO-EMF project office; (5) recent publication of the World Health Organization’s EMF project office (WHO-EMF) commissioned systematic reviews; and (6) finally, it includes some observations on an apparent paradigm shift.

## A historical perspective on RF exposure standards

Interest in the biological effects of RF radiation goes back toward the end of the 2nd World War. The ensuing development and research during the 1940’s and 1950’s have revealed that RF and microwave exposure can initiate both favorable and harmful biological consequences in humans by heating tissues in the body. The heating may or may not be sensed as temperature rises via simple temperature monitors. Nevertheless, the information was prominent in deciding 100 W/m^2^ (10 mW/cm^2^) of incident power density in 0.1 h as a safety standard for human exposure to RF and microwaves in 1966 (5, 6). Further examinations yielded a slight amendment to the standards in 1982 (9). The effort was due to a collaboration between what is now known as the IEEE and the U.S. Department of Navy. However, the available scientific data was only able to offer the rudimentary structure for a less than rigorous or precise exposure standard (i.e., adjusting the rough time limit from 0.1 h to 6 min). Hence, research on biological effects and safe use of RF and microwaves persisted and the pool of scientific knowledge gradually expanded.

An important part of the research was the quantitative estimation of the amount of absorbed RF energy inside the body needed for an observable biological effect induced by an incident power density. Introduction of the metric of specific absorption rate (SAR) and its frequency dependence on the incident power offered the foundation for determining a permissible maximum exposure. The findings formed the reasoning for reporting quantitatively results from laboratory observations (10). SARs are applicable to link the RF and microwave exposure to reactions observed from research studies. It can help to better and more quantitatively understand the biological phenomena. And it is not dependent on any interactive mechanism(s). The SAR can act as a scale marker for extrapolating experimental data from cells to tissue, tissue to animal, animal to humans, etc. Indeed, SAR can be used to assess associations among various observed responses in different investigational models and test subjects.

Additional progress has significantly supported the enhancement of existing exposure standards. For example, in its report on biological effects and exposure criteria for microwave and RF radiation (11), the U.S. National Council on Radiation Protection and Measurements (NCRP) recommended the exclusive use of SAR for quantification of RF and microwave distribution and absorption in biological materials or animal bodies under exposure. Consequently, SAR was used in the 1992 edition of exposure standards developed by the IEEE Standards Association, which was also recognized by the American National Standards Institute (12).

The continued spread of cell phones and mobile communication devices and systems and the persistent concerns about the health impact and safety of the ubiquitous RF and microwave radiation prompted the U.S. Federal Communications Commission (FCC) to publish in 1996 rules for permissible human exposure to RF and microwave radiation from cellphones and RF base stations (13). The FCC rules instituted for SAR are identical to the NCRP recommendations (1.6 W/kg for any 1-g of tissue mass) and they are also effectively the same as the voluntary ANSI/IEEE-1992 standards for the applicable frequency ranges. However, with one important distinction, the FCC rules are enforceable by law.

Globally and historically at that time, electromagnetic radiation protection guidelines were developed by the International Commission on Radiological Protection (ICRP) under the auspice of the International Radiation Protection Association (IRPA). The primary focus then was on ionizing radiation. In 1992, IRPA chartered a new International Commission on Non-Ionizing Radiation Protection (ICNIRP) with the objective of developing internationally accepted recommendations for nonionizing radiation. This was the culmination of a process initiated some years ago as more evidence of biological effects and health implications of microwave and RF radiation began to appear in scientific publications.

In 1998, ICNIRP published its recommended guidelines (14).

These guidelines closely resemble the ANSI/IEEE-1992 standards and FCC-1996 rules. However, ICNIRP elected to fix the SAR value at a high level of 2.0 W/kg averaged over 10-g mass of biological tissue for local absorption. It did so without any plainly described scientific rationale or biophysical basis.

The new name of International Committee on Electromagnetic Safety (ICES) was accepted in 2001 by the IEEE Standards Association to replace the previous bodies (IEEE C95.1 Committees), which developed the ANSI/IEEE-1992 standards.

The IEEE-ICES published a set of revised exposure standards in 2006 that deviates substantively from the 1992 ANSI/IEEE edition (and its interim modifications). Specifically, it adopted ICNIRP’s SAR value of 2.0 W/kg value as averaged over a 10-g tissue mass for local absorption (15). It is ostensibly done in a gesture aimed at global standards harmonization but not necessarily to acknowledge the contemporary advancements in the science of health safety protection.

In recent years, both ICES and ICNIRP have published revisions of their recommendations for exposure limits (16, 23). The revisions appear to accommodate industry or business-related objectives and are grounded on a strong RF heating conviction—only RF heating effects revealed by measurable tissue temperature rises. They are constructed on the base of biological data from short-term (less than 6 or 30 min) exposures. They do not propitiate the expressed lack of confidence in these RF exposure standards that are recurring in many parts of the world (17–22).

## Current safety standards and guidelines

A brief summary of the current ICES and ICNIRP recommended guidelines or standards for human exposure to RF radiation (16, 23) is given in Table 1. It shows that for frequencies between 6 GHz and 300 GHz including 5G cellular mobile communication, the exposure limits allow tissue temperature rises in the human head, limbs, and torso to as high as 5°C. This amount of rise would permit the tissue temperature to increase from a nominal 37°C to a hyperthermic 42°C. A hyperthermic tissue temperature of 42°C is cytotoxic with the potential for exponential cell kills. Furthermore, this level of hyperthermic temperature serves as the medical foundation for treatment of malignant tumors in hyperthermia therapy for cancer (24–26). The revised standards and guidelines offer a reduction factor of 10 for ordinary people at 20–40 W/m^2^ or a safety factor of 2 in workplaces from 100 to 200 W/m^2^. Under these scenarios, the effectiveness and safety of these limits is marginal, and they may be irrelevant from the viewpoint of health safety protection. They become especially troublesome when coupled with the knowledge of biological variability and measurement uncertainty.

**Table 1:** IEEE-ICES and ICNIRP recommended guidelines or standards for human exposure to RF radiation based on thermal effect (16, 23).

Frequency range	Tissue type	Temperature rise	Average mass	Average time	Health effect level	Factor^b^	Public level	Factor^b^	Worker level^c^
100 kHz–6 GHz	Local head-torso	2°C	10 g	6 min	20 W/kg	10	2 W/kg	2	10 W/kg
	Local limbs	2°C	10 g	6 min	40 W/kg	10	4 W/kg	2	20 W/kg
>6 GHz–300 GHz^a^	Local head-torso	5°C	4 cm^2^	6 min	200 W/cm^2^	10	20 W/cm^2^	2	100 W/cm^2^
30 GHz–300 GHz^a^	Local limbs	5°C	2 cm^2^	6 min	400 W/cm^2^	10	40 W/cm^2^	2	200 W/cm^2^
100 kHz–300 GHz	Body core	1°C	WBA^d^	30 min	4 W/kg	50	0.08 W/kg	10	0.4 W/kg

^a5G, bSafety or Reduction Factor, cControlled or Occupational Exposure, dWhole-body Ave – WBA.^

## Assessment of revised limits for health safety protection

A recent paper challenged the health safety offered by the current exposure limits to RF radiation and called for an independent evaluation of the scientific evidence (18). It unveiled that the existing exposure limits disregarded many scientific papers which document harmful biological responses at exposure levels below the threshold asserted by these safety guidelines. It further argues that the scientific data invalidates the health suppositions underlying the pronounced RF exposure restrictions. Specifically, for frequencies below 6 GHz, a SAR value of 4 W/kg, temporally and spatially averaged over the whole body was assumed as the effective threshold for adverse health effects in humans. The level was predicated on the disruption of operant-conditioned-work schedules in a few trained rodents and primates (27–29). The current limits assume a heat production rate of 4 W/kg is within the normal range of human thermoregulation. They are introduced to prevent only harmful thermal effects on human body functions.

It is noteworthy that ICNIRP was concerned about the biological effects of pulsed RF and microwave signals in its 1998 guidelines but have mysteriously dropped them from the 2020 recommendations. Accordingly, any specific pulse modulation, key to cellular mobile communication technologies, is regarded the same as sinusoidal or continuous wave (CW) signals. The time-averaged SAR over a 6-min period is woefully inadequate to account for the unique characteristics of pulse modulations (with nano to micro-second pulse widths) or to capture the effects of pulse modulated exposures including the microwave auditory effect, which occurs for microsecond pulses without any measurable temperature rise and at low levels of SAR (30–32).

The applicability of the limits for safe long-term exposure to low-level RF radiation is questionable. The revised exposure limits do not provide any adjustments for or protection against effects due to long-term human exposures. There is palpable lack of appreciation of scientific knowledge for chronic toxicity and carcinogenicity regarding RF exposures below the basic restrictions promoted in the existing exposure guidelines and standards (17–22, 33).

Regarding epidemiological studies of cell or mobile phone RF radiation and carcinogenicity, the recent revisions (16, 23) claim that results on gliomas, meningiomas, parotid gland tumors, and acoustic neuromas (vestibular schwannomas) have not offered the proof of an increased cancer risk. Also, the revised recommendations and standards opined that while there are reports of greater odds ratios, methodological differences and imagined weaknesses including putative recall and selection bias thwarted the epidemiological results from being considered for the recommended guidelines. The penchant to criticize and deny positive results, and passion for and keenness to accept negative findings, concurrently, are palpable and troubling. They are aiding and abetting the causes for the expressed lack of confidence in the current RF exposure guidelines and standards worldwide.

### Denial of IARC, NTP and Ramazzini findings

The International Agency for Research on Cancer (IARC)—an intergovernmental agency of the World Health Organization (WHO) classified exposure to RF radiation as 2B—a possible cancer-causing agent in humans (34, 35). A classification of possibly carcinogenic to humans (Group 2B) is third on IARC’s five categories of carcinogenic risks. The highest category (Group 1) is reserved for biological, chemical and physical agents that are carcinogenic. It is followed by Group 2A—probably carcinogenic, Group 2B—possibly carcinogenic, and Group 3—not classifiable as to its carcinogenicity; and lastly, Group 4—probably not carcinogenic to humans.

The responsibilities of IARC are to coordinate and conduct health related research into the causes of cancer in humans. It evaluated the available scientific investigations and concluded that epidemiological observations in humans displayed higher risks for the glioma type of malignant brain cancer and benign acoustic neuromas (i.e., vestibular schwannoma of the vestibulocochlear nerve) among heavy or long-term users of cell or mobile phones. The evidence is sufficiently robust to underwrite a classification of RF radiation from cell and mobile phones as a possible cancer-causing agent for humans. In the meanwhile, it recognized the existing database was imperfect and limited, especially regarding laboratory results from animal experiments.

Significantly, the results from animal experiments that was lacking were later provided by the U.S. National Toxicology Program (NTP) report of two types of cancers in laboratory rats that were exposed, lifelong, to 2G and 3G cell-phone RF radiation frequencies below 6 GHz (36–38). This carcinogenic investigation was the largest health effect animal study undertaken by scientists at NTP. It is also the biggest animal health research done using cell or mobile phone RF radiation exposure as the independent experimental variable. The results showed clear and statistically significant indication that RF radiation was the cause for the observation of a rare form of malignant cancer (schwannoma) inside the heart of male rats. The animal’s body temperature rise induced by RF exposure did not exceed 1°C at the highest exposure (SAR = 6 W/kg). There was some suggestion of a schwannoma risk among the females. The study also observed injuries inside the heart (cardiomyopathy) due to RF exposure in both males and females compared to concurrent control rats. In addition, the pathological outcomes based on statistical significance exhibited signs for RF radiation dependent carcinogenic activity (gliomas) in the brain of male subjects. The observations from females were judged as equivocal for malignant gliomas compared to concurrent control rats.

The NIH-NTP project is by far the largest NTP animal cancer study, including toxic chemical agents (39, 40). The lifelong (2-year) exposure study of rats subjected to 900/1,900 MHz RF radiation involving GSM and CDMA cell and mobile phone operations. GSM and CDMA are commonly used by 3G wireless mobile telecommunications technology.

It is important to note that following a thorough review of the RF animal exposure study, pathologists and toxicologists on the NTP External Peer Review Panel on 28 March 2018 concluded, among others, there is statistically significant and “clear” evidence that both GSM and CDMA-modulated RF exposure had resulted in the development of malignant schwannoma, a rare form of tumor in the heart of male Harlan-Sprague–Dawley rats, and there was “equivocal evidence” for the same schwannoma risk among female rats. The panel also observed that there were unusual patterns of cardiomyopathy, or damage to heart tissue, both in RF-exposed male and female animals compared with concurrent control rats.

In addition, the panel decided based on statistical significance that the pathology findings gave clues of “some evidence” for RF exposure-dependent carcinogenic activity in the brain of male rats (glioma). However, the findings for female rats were deemed as providing only “equivocal evidence” for malignant gliomas compared with concurrent controls (37).

The 14-member external peer review panel consisted of 10 pathologists and toxicologists (3 from universities and 7 from industry), 3 electrical engineering professors, and one biostatistician; none were employed by or connected to the cell or mobile phone industry.

The Ramazzini Institute in Bologna, Italy reported, in that same year, the results from its large rodent study of cancer risks in rats exposed to 3G cell or mobile phone RF radiation using established good laboratory practices (GLP). The research involved whole-body exposure of the same strain of rats as NTP, either lifelong or prenatal until death, under far-field plane wave exposure conditions (41). During the 19-h-day for roughly two-year exposures, the calculated whole-body SARs were 0.001, 0.03, and 0.1 W/kg. A statistically significant elevation in schwannomas in the male rat hearts was documented for the 0.1 W/kg RF exposure. The fact that the NTP and Ramazzini studies delivered similar effects for heart schwannomas and brain glioma is an important finding. Specifically, two well-conducted large RF animal studies including life-long exposures of the same rat strain disclosed consistent carcinogenicity effects.

Apparently, the positions taken by the recent safety limit revisions on these animal results are denials. They chose to utterly ignore the independent variable for the experiments, i.e., RF radiation from wireless communication devices and systems. Instead, they opted to object with self-assumed “chance differences” in the experiments or complications of induced body-core temperature rises to 1°C in rats at the highest (6 W/kg) RF exposure levels. In doing so, it bizarrely overlooked the absurdity of proposing a 1°C body-core temperature rise as a cause for cancer. Perhaps, it was an ill-guided attempt to dodge using subterfuges such as the “findings do not provide credible evidence of adverse effects” produced by chronic RF exposures. Vague expressions such as “substantial limitations” were deployed to defend the motives in preventing any “conclusions being drawn concerning RF exposures and carcinogenesis,” and to rationalize the recommended RF limits.

The results from laboratory animal experiments should help to update and elevate the IARC classification to the carcinogenic category or at least elevate it to the next higher level as probably carcinogenic. Nonetheless, the revised recommendations evaded them by irrationally declaring the experimental laboratory findings do not provide credible evidence of adverse effects induced by chronic RF and microwave exposure.

Note that in a recent study (42) the NTP and Ramazzini researchers collaborated to assess the genetic modifications in RF-induced rat tumor samples through molecular characterization of cancer genes related to human glioma genesis. A targeted next generation sequencing (NGS) panel was constructed for rats based on human glioma-relevant genes. Single-nucleotide variants and small insertions and deletions were categorized in the rat gliomas and cardiac schwannomas. The results indicate that rat gliomas induced by life-long RF exposure histologically appear like low grade human gliomas.

It is interesting to note that recently the IARC Advisory Group has prioritized reevaluation of the carcinogenicity of RF radiation within the next 5 years (43). While any change in the current IARC classification of the carcinogenicity of RF radiation must await, it is well for IARC to keep in sight the recent report that rat gliomas resulting from life-long RF exposure histologically resemble low grade human gliomas (42).

### The standards are less precise and affect only short-term RF exposures

A topic of concern is the development of exposure standards and guidelines on the basis of flawed conjectures rather than scientific evidence. The case may be discerned from the harmonization of IEEE’s SAR limit value of 1.6 W/kg for a 1-g tissue mass to ICNRIP’s 2.0 W/kg for a 10-g mass during short-term exposures below 6 GHz. In the 1980’s, the adoption of SAR as a dosimetry quantity and establishment of the value 1.6 W/kg for a 1-g mass were examined with great scientific deliberation with sensibility and they were reaffirmed through several renditions of IEEE-ICES standards in the early 2000’s. The decision by ICNIRP in 1998 to choose the SAR value of 2.0 W/kg was not accompanied by any stated scientific rationale or biophysical basis. Global harmonization of RF and microwave exposure standards and guidelines would be a desirable goal. However, it should not be approached unconvincingly on a basis of harmonization for harmonization’s sake. The procedure should point to advances beyond the current state-of-affairs, through better precision in SAR specification and lower uncertainty in exposure assessment.

In December 2019, FCC reaffirmed its RF exposure rules (44). The regulatory action proceeded notwithstanding the many appeals. Some propose relaxing the rules, and others to tighten them. Among promoters to weaken the rules are suggestions from consultants for the wireless industry, CTIA—The Wireless Association, Mobile Manufacturers Forum (MMF), and Telecommunications Industry Association. Those appeals also argued that the evidence for health effects imply 5G is akin to any other installed cell or mobile technology and systems. Claims were presented for lessening cell-phone RF rules to ICNIRP’s 2.0 W/kg SAR over 10 g of tissue, a less precise measure, instead of the FCC’s regulation at 1.6 W/kg over 1 g.

Aside from the obvious 25% numerical increase of SAR value from 1.6 to 2.0 W/kg, the enlargement of averaging tissue mass from 1 g to 10 g substantively reduces by 10-fold the accuracy of SAR calculations. Thus, the harmonization scheme could have a combined impact of raising or relaxing the permissible IEEE exposure limit or FCC rules by a factor of 250%—a significantly lower safety protection! Furthermore, it is important to keep in mind the vast differences of biological issues in cell types, quantity and variety in a 1-g or 10-g mass of living tissues.

Research on correlation of SAR and induced tissue temperature elevation revealed a close dependence on size of averaging tissue mass and exposure duration (45). The investigation involved anatomically realistic models of the human body. It studied the impact of averaging mass and SAR on the correlation between RF energy and induced tissue temperature elevation. It found that SAR provides a better correlation with temperature for short exposures. For the frequencies investigated (700–2,700 MHz), the best correlation with temperature rise happens for exposure periods between 1 and 2 min for SAR. In this case, a mass of 1 g is optimal for correlation of temperature elevation with SAR. For longer exposures, the correlation is reduced in favor of larger averaging mass. At steady-state exposures greater than 30 min, the correlation of temperature increase with SAR is maximum for a mass of 9 g (~10 g) for the frequencies considered. In science-based exposure standards and guidelines, the applicable averaging mass for frequencies below 6 GHz should not be the same for short-term and longer exposure durations even for heating-related safety standards or guidelines.

In brief, the revised RF exposure standards and guidelines account only for tissue heating with RF radiation. The recommendations are formulated to prevent short-term heating. They are flawed and are not applicable to long-term, low-level exposures. Instead of advances in science, they are limited by misguided exposure metrics that do not adequately protect children, workers, and the public from exposure to RF radiation or people with sensitivity to RF electromagnetic radiation. The recommendations bypass notable laboratory animal results. They disregarded conclusions by scientific organizations such as IARC. Many of the recommendations are disputed and are absent of scientific justification from the perspective of safety and public health protection. They fail to manage the health risks by not adhering to the three important ICRP principles of radiation protection: justification, optimization, and ALARA—As Low as Reasonably Achievable (46).

## An industry-regulatory complex

The termination of NIH-NTP’s RF effects research program on how RF radiation causes cancer essentially halted all nonmilitary biological research of RF and microwave radiation underwritten by the U.S. government (47, 48). Concurrently, the efficacy of publicized health safety rules, standards and recommendations for RF radiation used by the wireless cellular mobile communication devices and systems has become major concerns. Questions are being raised about the U.S. government’s seriousness regarding scientific research on health and safety of RF radiation with the halting of the relevant research programs.

Perhaps, the relation between U.S. regulatory agencies and telecommunication industry might be paraphrased as an “industry-regulatory complex,” a networking effort to attain unwarranted influence and power, and for continued or increased regulatory relief or support of the industry. It may include bringing major industry actors into positions of power in government that regulate those industries in the fashion of a revolving door. An industry regulatory complex aimed at promoting policies that may not be in the general public’s best interest and its growing impact, if left unrestrained, could potentially undermine public health and safety.

In 1968, the U.S. Congress authorized the Radiation Control for Health and Safety Act. The debates before emphasized the paucity of scientific knowledge on the biological effects and health implications of exposure to both ionizing radiation such as X-ray and nonionizing radiation such as microwaves. The discussions uncovered the substantial amount of avoidable radiation people were being exposed to. Congress had proclaimed that the public’s health and safety must be protected against the hazards of radiation from electronic products, including microwaves. The act empowered the federal government to establish radiation standards, check compliance, and conduct research. It directed the U.S. Department of Health, Education, and Welfare (HEW) to set up and execute an electronic product radiation control system devised to protect the public’s health and safety from radiation produced by electronic products. To comply through its Food and Drug Administration (FDA)’s Bureau of Radiological Health (BRH), the FDA set a federal standard (21 CFR 1030.10) that restricts the quantity of microwaves that may emit from a microwave oven during its time in service to 5 mW of microwave radiation per square centimeter (5 mW/cm^2^ = 50 W/m^2^) or 194.17 volts per meter (V/m) at 5 cm, approximately 2 in. from the oven surface. Note that the Federal Communications Commission (FCC) set forth the same rules for maximum permissible exposure (MPE) to the relevant microwave and RF radiation but without the distance specification (44). Thus, the FCC relaxation on the spatial condition could imply unsafe exposures according to FDA’s microwave oven performance standards. It should be noted that HEW was retitled as the Department of Health and Human Services (HHS) in 1979 to show its new mandates, without the education portfolio. The Center for Devices and Radiological Health (CDRH) has replaced BRH.

In 1999, FDA nominated RF and microwave radiation exposure associated with wireless communication devices and systems, especially cellular mobile phone-related exposures for toxicology and carcinogenicity testing in animal models by the US National Institutes of Health’s National Toxicology Program (NIH-NTP). The nomination was prompted by factors such as the prevalent global usage of cellular mobile communications among users of all ages and consideration of the lack of clear statistics from epidemiologic studies in humans and inconsistent findings from experimental studies in animals. After extensive evaluation of the published literature and experimental investigations at the time, the NTP resolved that additional laboratory studies were warranted to define any potential health hazard more clearly to humans from long-term cell-phone microwave and RF radiation exposure (37). It is noteworthy that NTP’s mandates are to provide the scientific basis for U.S. programs, activities, and policies that promote health or lead to the prevention of disease. Its operational axiom is “science you can depend on for decisions that matter.” NTP’s results are widely regarded as the gold standard for studies of animal toxicology and tumorigenesis.

While the FDA acknowledges that the NTP study is the most comprehensive study of the impact of microwave and RF radiation on tumorigenesis to date, it does not agree with all the conclusions of the NTP study (49). Specifically, it decided that “we cannot draw conclusions about the impact of such exposure to humans based on these *in vivo* animal studies.” Furthermore, it lamented that “if there had been even one case in the control group then the statistical significance of this finding would not exist.”

The Telecommunications Act of 1996 had massive influences on the telecommunications and internet industry across the United States, including competitiveness and consumer protection. While it enabled market concentration in the media and telecommunications industries, the Act has been ineffective at promoting public health and safety (17). For example, in response to lawsuits filed by the Environmental Health Trust and other groups, the U.S. Court of Appeals in Washington, D.C. ruled in August 2021 that the FCC had failed to meet “even the low threshold of reasoned analysis” in concluding that its limits “adequately protect against the harmful effects of exposure to RF radiation,” including those from cell phones (50). Furthermore, the court ruled that the FCC failed to adequately address comments and concerns regarding the adequacy of its guidelines for exposure to RF radiation. The court found that the FCC did not provide an explanation for its decision to terminate a notice of inquiry regarding potential modifications to its limits.

The Act directs the FCC to evaluate the scientific evidence in support of its regulatory rules and assess the impacts of its actions on the quality of the human environment, including human exposure to microwave and RF radiation emitted by FCC-regulated sources and facilities. FCC is a dedicated technological expert agency without in-house health or medical expertise of its own. In establishing its health and safety rules it consulted the FDA, an expert agency regarding the efficacy of medical devices and charged with regulating the health and safety impacts of consumer products. The FCC issued its rules in 1996, which set limits for RF exposure it believes reflecting the available information regarding safe levels of RF exposure for workers and members of the general public (51).

The resistance of the cell-phone industry is understandable. It would be damaging to their business model if their products were associated with negative consequences for public health. However, cavalier refusals from the authorities responsible for health and radiation protection are untoward and concerning.

The success of the U.S. military-industrial complex in shaping research regarding the biological effects from exposure to microwave and RF radiation and their influence on setting of health and safety standards for humans are widely known (52). Perhaps, the network between U.S. government and telecommunication industry might be similarly termed or paraphrased as an “industry-regulatory complex.” They appeared to have spared little efforts through networking to marshal political influence and power and for continued or increased regulatory relieve of the impacted industries. In this case, a revolving door that brings major industry players into positions of power in government that regulates those industries should not escape notice. An industry-regulatory complex aimed at promoting policies that may not be in the country or citizens’ best interest could undermine public health and safety.

A case in point, on May 1, 2013, President Barack Obama nominated Thomas E. Wheeler (Tom Wheeler) as chairman of the FCC. At the time, many people have voiced apprehension on the thought of Wheeler for the prospective appointment due to Wheeler’s history of lobbying for the industry, which the FCC is responsible in regulating. He was confirmed nonetheless by the U.S. Senate in November 2013 and served until January 20, 2017. Among his other industry roles, Tom Wheeler led the industry’s main trade group Cellular Telecommunications & Internet Association (CTIA) from 1992 to 2004. During that period, Wheeler initiated the industry’s $25 million-dollar, decade long, privately run research program intended to reassure the public that cell phones were safe (53).

Works in the U.S. to promote awareness of microwave and RF-radiation risks had generated intense opposition from industry. In 2014, the Centers for Disease Control and Prevention (CDC) included some modest words to its website: “Along with many organizations worldwide, we recommend caution in cellphone use.” A powerful industry consultant emailed the CDC within days, complaining that “changes are truly needed.” A public records request subsequently revealed. The agency promptly softened its warning to say: “Some organizations recommend caution in cellphone use” (17).

In recent years, CDC has funded a congressionally chartered private sector entity—the National Council on Radiation Protection and Measurements (NCRP) under its SC8-1 committee to develop informational webpages on the use of wireless technology and other related health effect matters (54, 55). Apparently, the CDC is concerned that information posted on some websites may sow misgiving and undermine assurance in federal health and safety standards that were established putatively to protect the public from harmful exposures of microwave and RF radiation. The issues to be covered include subjects such as the controversy and relevance of low level biological effects, i.e., other than the “accepted” thermal effects, as they pertain to microwave and RF emissions from wireless technology.

Curiously, only two out of the nine members of NCRP SC 8-1 committee on Development of NCRP Informational Webpages to Provide Authoritative Information About the Use of Wireless Technology and Current Evidence on Health Effects (55) have expertise in RF effects or wireless technology radiation. And both of them have frequently served as industry consultants. Those members have repeatedly articulated their strong convictions that there is nothing but thermal effects resulting from rises in tissue temperatures to worry about with microwave and RF radiation. In addition, the chair of the SC8-1 committee appointed to lead the CDC-NCRP nonionizing informational webpage development was criticized as “not particularly well versed in RF” as the public records request revealed. The same person has questioned the risk of harm from the electromagnetic fields (EMF) of power lines and regards fears of it are unfounded. These circumstances would do little to placate the troubling question on the lack of confidence in the microwave and RF exposure guidelines that persist in many parts of the world.

## WHO-EMF’s systematic reviews

The World Health Organization’s EMF Project (WHO-EMF) has recently published several of its commissioned systematic reviews on health effects of microwave and RF radiation from cell and mobile phones and wireless communication devices and systems. The reviews are published as part of a special series in the journal Environment International, with Paul Whaley as the handling editor.

Apparently, at a Navigation Guide workgroup webinar, hosted by University of California at San Francisco on December 17, 2021, Whaley presented on the planned reviews as a WHO-EMF team member alongside Emilie van Deventer, Martin Röösli, and Jos Verbeek.

The WHO-EMF project was established to evaluate environmental and health effects of exposure to electromagnetic fields and radiation in the spectral domains of 0–300 GHz. While it claims that its funds are received by payments from WHO member states, it does not release what portions of WHO-EMF finances come from government or industry sources (56). Indeed, it acknowledged that up to 50 percent of the funds raised for the WHO-EMF project came from industry sources and that other contributors have provided staff time. The staff time appears to have been comprised of people with connections to ICNIRP. Note that a major source of funding for ICNIRP comes from the German Federal Office for Radiation Protection (57).

The protocols for the WHO-EMF systematic reviews (SR) were released starting 2021 (58–60). Each review followed a detailed protocol. Generally, the SR study methodology utilizes a protocol for grouping, assessing and summarizing all relevant published studies on a research subject. The methodology encompasses defining the subject, deciding inclusion and exclusion criteria, evaluating the quality of the studies, analyzing the data, and reporting the results. However, failures such as improperly evaluated quality of study or data in SRs could steer toward misleading inferences. Accordingly, various recommendations have been suggested for conducting SRs to assist in enhancing the scientific value, consequence and utility of SRs. The objective is to mitigate the likelihood of unexperienced reader may be misdirected in the subject matter. Accepting the conclusions of a SR without proper appraisal to ascertain its veracity, limitations, transparency and credibility can be precarious where public health policy is concerned. For example:

(1) The WHO-EMF systematic review on the association between RF exposure and adverse health effects pertaining to reproductive health (pregnancy and birth outcome) concluded that in utero RF exposure does not have a detrimental effect on fecundity but likely affects offspring health at birth (61). Regarding a possible late effect of in utero exposure, RF and microwave radiation probably does not affect offspring brain mass and may not decrease female offspring fertility. While RF and microwave radiation may have a detrimental impact on neurobehavior functions, these findings are very unreliable.

A detailed assessment of the quality of this SR and evaluation of the relevance of its conclusions to pregnant women and their off springs shortly followed in a peer-reviewed publication (62). The quality and relevance were tested using the review’s collection of papers and chosen statistical methods. While the WHO-EMF SR reports itself as thorough, scientific, and relevant to human health, numerous issues were identified rendering the WHO-EMF SR severely flawed and irrelevant. The flaws found skewed the results in favor of the review’s conclusion that there is no conclusive evidence for effects other than RF induced tissue heating. In fact, this paper showed that the underlying data, when relevant studies are correctly cited, support the opposite conclusion: “There are clear indications of detrimental nonthermal effects” from RF exposure. The authors identified a multitude of flaws which enabled them to uncover a pattern of systematic skewedness that appeared to aim for uncertainty hidden behind complex scientific rigor. To those scientists the skewed methodology and low quality of the systematic review was highly concerning, “as it threatens to undermine the trustworthiness and professionalism of the WHO-EMF project in the area of human health hazards from human-made RF radiation.”

(2) The WHO-EMF SR of human observational studies (63) on the occurrence of migraine, headaches, tinnitus, sleep disturbances and non-specific symptoms in the general and working population stated that the body of scientific evidence reviewed supports the safety of currently promulgated ICNIRP guidelines for RF exposure (16).

An ensuing critical appraisal by three accomplished senior researchers documented major problems with the WHO-EMF-commissioned review and called for its retraction (64). The meta-analysis for the handful of very diverse primary studies associated for the analyzed exposure and outcome combinations is profoundly improper. The number is very small, and the methodological attributes of the relevant primary studies are low. In contrast, this peer-reviewed critique concluded that the body of evidence reviewed is incapable of either supporting or refuting the safety of recent exposure recommendations.

(3) Skepticisms have been expressed regarding a third WHO-EMF systematic review on RF–induced oxidative stress (65). The study identified 11,599 papers on oxidative stress in the spectral domain of 800–2,450 MHz and then rejected 11,543 of studies as not meeting the criteria for inclusion. Of the remaining 56 papers, there are 45 animal studies and 11 *in vitro* cellular investigations. The outcome was that a majority of the remaining studies displayed high heterogeneity. The oxidative stress responses were inconsistent across the experimental subjects studied. There may or may not be a response to RF and microwave exposure, but the certainty of the data is very low.

For many years, Henry Lai, a well-known researcher in RF oxidative responses, has maintained a bibliography of RF-oxidative stress papers (47). As of August 2024, his file consists of 367 papers, published between 1997 and 2024 and 89% of them showed significant responses. His assessment of the WHO-EMF review is that it left out a large portion of RF-oxidative studies and appears basically only considered oxidative molecular reactions among the possible oxidative responses. As noted earlier, this systematic review appears to have methodically excluded most of the relevant research, about 99.5 percent.

(4) The WHO-EMF systematic review on human epidemiological studies comes with a subtitle of “Most Researched Outcomes” (81). The purpose of this SR was to assess the quality and strength of the evidence provided by human epidemiological studies for a causal association between RF exposure and risk of the most investigated neoplastic diseases. The study selected 63 papers, published between 1994 and 2022. It concluded that RF exposure from cellular mobile phones was not associated with an increased risk of glioma, meningioma, acoustic neuroma, pituitary tumors, or salivary gland tumors. The conclusion suggested that there was not an observable increase in relative risk for the most investigated neoplasms (glioma, meningioma, and acoustic neuroma or vestibular schwannoma) with increasing time since the start of cellular mobile phones, cumulative call time, or cumulative number of calls. The message is clear—there is little evidence to justify continued concern over a possible cancer risk. This WHO-EMF SR was reported on by many Western media outlets. Actually, there is truly little data that is new in this review. For sure, assessment of scientific evidence in this subject has been controversial and less than uniform. The inevitable question—“is this review really the definitive word on the long-standing issue of whether cell phone radiations pose a cancer risk?” The answer is resounding no, far from it!

The Microwave News (66) published a meticulously researched investigative report in the historical context of the WHO-EMF cancer SR. “This is just the latest gambit by the usual suspects at ICNIRP and the WHO that have been making similar claims for the last 20 years.” Five years ago, the lead author (81) with some members of the same team made similar efforts to terminate the RF-cancer debate with basically the same no-risk message. However, “it was not well received” by the scientific community. Evidently, the analysis excluded some people older than 59 years of age from the analysis—the largest segments of the brain cancer population. The decision essentially predestined the no-risk result.

It is well to recall IARC in 2011 classified exposure to RF radiation as a possible carcinogen in humans on the strength of human epidemiological evidence (34). The IARC agreed that the epidemiological observations in humans exhibited higher risks for the glioma-type of malignant brain cancer and a benign vestibular schwannoma of the vestibulocochlear nerve among heavy or long-term users of cellular mobile phones. It deemed that the epidemiological evidence was satisfactorily robust to justify the classification. As described earlier, subsequent results from animal experiments provided by the NIH-NTP showed two types of cancers in laboratory rats that were exposed, lifelong, to cell-phone RF radiation (37, 38). The research finding was complemented by another well conducted, large RF animal exposure study involving life-long exposures of the same strain of rats in the same year from the Ramazzini Institute in Italy (41).

The WHO-IARC, NIH-NTP, and Ramazzini outcomes, under normal circumstances, would likely have provided the justification for raising WHO-IARC’s current possible cancer risk designation to the probable cancer-causing classification, if not higher. In this regard, it is noteworthy that in a recent study the NTP and Ramazzini researchers examined the genetic alterations in RF-derived rat tumors through molecular characterization of cancer genes relevant for human glioma genesis (42). The data suggest that rat gliomas resulting from life-long RF exposure histologically resemble low-grade human gliomas.

A most recent publication from the WHO-EMF commissioned SRs reviewed the effects of RF exposure on cancer in experimental animals (67). It included all 52 reported studies with 20 chronic bioassays. None of the reported studies were excluded from the SR in order to minimize the risk-of-bias concerns. Elevations in incidence or risk of two tumor types were identified in this systematic review. Specifically, an increase in glial cell derived brain cancer was reported in two life-long bioassays in male rats. The certainty of the evidence for an increased risk in glioma was judged as high. Also, in three chronic bioassays, statistically significant increases in malignant schwannomas were demonstrated as high in the heart of male rats. While this conclusion is in opposition to the interpretations of ICNIRP and perhaps, WHO-EMF itself, it is consistent with the findings of NIH-NTP, Ramazzini, and Brooks et al.

The criticisms and challenges encountered by the published WHO-EMF systematic reviews, aside from the most recent one, are serious and severe, including calls for retraction. Examinations of the reviews reveal major problems. In addition to the scientific quality of the less than balanced reviews, they appear to be biased with strong conviction of nothing but heat to worry about with RF microwave radiation. The unsubtle message that cellular mobile phones do not pose a cancer risk is clear. These systematic reviews exhibited a lack of concerns for conflict of interest and display unequivocal support for the recently promulgated ICNIRP RF exposure guidelines for human safety.

From its inception, WHO-EMF had close ties with the ICNIRP—a private organization, frequently referred to as the WHO-EMF project’s scientific secretariat (68). What may not be as apparent for most of the WHO-EMF systematic reviews is the lack of diversity of views and the nested opportunity for a groupthink mindset. A large number of ICNIRP commissioners and committee members, with varying levels of expertise, are listed as authors for the WHO-EMF systematic reviews, some also served as lead authors. The concerns exacerbate issues of reviewer independence and the potential for conflicts of interest in general.

## A paradigm shift

The U.S. Air Force Research Laboratory reported that whole genome bisulfite sequencing immediately following RF exposure showed changes in deoxyribonucleic acid (DNA) methylation patterns and early differentially methylated genes in human skin keratinocytes (69). The result highlights a possible epigenetic role in the cellular response to RF radiation. The report further suggested that the findings may potentially be developed as epigenetic biomarkers for immediate responses to RF exposure.

DNA methylation is an epigenetic process used by cells to regulate gene expression. It is dynamic and can be triggered in response to external stimuli such as ultraviolet (UV) exposure. The investigation exposed cultured human keratinocytes to 900 MHz RF radiation for 1 h at a low SAR (<0.01 W/kg), under the environmental conditions of 37°C, 5% CO^2^, and 95% humidity in a customized exposure system. The threshold for safe RF effects is 4 W/Kg per current standards. Six common targets were identified to both have differentially methylated and expressed in response to RF exposure. The specific process involved correlating global gene expression to whole genome bisulfite sequencing. (The investigation also identified 114 genes that were significantly differentially methylated immediately following a single 1-h RF exposure.)

Beyond underlining a potential epigenetic role in the cellular response to low-level RF exposure, these results place a spotlight on an atypical event, a paradigm shift in which a scientific investigation from an U.S. military research laboratory reporting a cytogenetic response (52). More specifically, it suggests an epigenetic role in the cellular response to low-level RF exposure, potentially, with major influences on gene activities.

Another example—the U.S. Army and Air Force Research Laboratories (70), recently conducted a computer simulation study of microwave auditory effect in an anatomical human head using the same approach employed in previous numerical studies (30, 32, 71–73). The computer simulation showed that for 1-GHz high-power microwave pulse exposures substantial acoustic pressure may occur within the brain that may have implications for neuropathological consequences (70). The simulation results were compared to previously established mechanically induced injury pressure thresholds for strain and stress associated with traumatic brain injury. The report showed the microwave exposures required are 10 and 15 W/m^2^ of peak power density for a 5-microsecond, 1-GHz pulse to reach the same threshold pressures of 10 and 20 kPa, respectively for explosive blast brain and football head impact injuries.

Although the required peak power densities are high, they are achievable with existing high-power commercial and military microwave systems operating under pulsed conditions (70). The disclosure comes as somewhat of a surprise to some, although it has been stipulated previously (30–32). Significantly, they also fall within the permissible “safe” limits of currently promulgated safety standards and protection guidelines. The required microwave technology is mature and in general, commercially available in many countries.

Furthermore, the study showed that to generate tissue injuring level of high-power microwave induced acoustic pressure waves inside the human brain, the microwave pulse induced temperature elevation would be substantially below the assumed threshold for RF effects (1°C) which is again considered “safe.” Therefore, the exposure would be allowable according to currently promulgated RF and microwave safety protection guidelines.

In 2017, the U.S. Defense Advanced Research Projects Agency (DARPA) announced a new research initiative: RadioBio: What Role Does Electromagnetic Signaling Have in Biological Systems? (74, 75). The objective of this project was to establish if purposeful signaling via electromagnetic waves between biological systems may exist, and, if it does, find ways to define what information is being transferred.

The goal of RadioBio is innovative and the project is intriguing. They seem also to suggest a paradigm shift in the U.S. military’s standard of operation procedures (SOP), away from a conviction of nothing but thermal effect could be associated with electromagnetic fields and waves. The new initiatives instead appeared to allow exploration (and perhaps exploitation) of low-level, nonthermal biological responses to RF exposure.

Furthermore, the RadioBio initiatives are beginning the process to actively search, ascertain, and study the potential role of low-level electromagnetic fields and waves could possibly have in the intricate biology of living cells and organisms. The initiatives are of not only fundamental scientific importance, but they also conjure up practical and technological significance. The possibilities and potential applications in data transfer, information delivery and retrieval, communication, and sensing for command and control are enormous, once the bioelectromagnetic mechanisms for weak cell-to-cell signaling and communication in living organisms are harnessed.

Perhaps, the recent publications from some of the military research laboratories may serve as telltales of more to come. A case in point is the reporting of detection of RF radiation from the microorganism *Staphylococcus aureus* (*S. aureus*) in biofilms (76) and its follow-on paper (77). The study is apparently funded by DARPA’s RadioBio program.

Note that RF and electromagnetic field interactions with biofilm-associated microorganisms and *S. aureus* have been reported (78, 79). Specifically, exposure to modulated electromagnetic fields and mobile communication RF (Wi-Fi) signals were shown to influence the response of biofilm bacteria leading to alterations in expression of messenger RNAs and morphological changes. Biofilms or bacterial biofilms are comprised of microorganisms such as *S. aureus* or *Escherichia coli* (*E. coli*) in which cells stick to each other and attach to and grow on surfaces. These adherent cells produce and form extracellular matrices of polymeric substances that result in altered phenotype of the organisms with variable growth rate and gene transcription.

The RadioBio funded papers reported successful detection of RF radiation from *S. aureus* biofilm in the 3.18 GHz and 3.45 GHz frequency bands via a radiometer type of detector. Both short-term and long-term variations of the radiation intensity were observed. To demonstrate that the RF signals are indeed produced by living cells, a lethal dose of Zinc oxide nanopyramids (ZnO-NPY) was administered to the sample. The results showed drastic reduction in RF intensity variations of detected signals before and after ZnO-NPY treatment. This observation is essential in demonstrating the viability of *S. aureus* biofilm for the detected RF signals. However, the genesis, nature or source of detected RF signal is obscure. It begs the question of how is the detected signal related to activity of the living bacteria biofilms? The records do not preclude the consequences of dynamic events taking place within the living bacteria biofilms which may be construed as signals instead of artifacts.

The analogous experiments where the RF intensities measured from peptone NaCl glucose (PNG) media with biofilms (biofilm samples) were compared with that measured from fresh PNG media void of biofilms (PNG samples) are interesting. The many orders of magnitude difference in measured intensity levels between the biofilm samples and the PNG samples are unremarkable. It has been shown that *S. aureus* biofilms grown in PNG medium are more resistant to disassembly and degradation (80).

In another set of experiments, a sinusoidal signal at the RF frequency of 3.18 GHz was used to expose the biofilms. The biofilm samples were reported to exhibit stronger RF-related characteristics after being exposed to 3.18 GHz radiation. Furthermore, a similar experiment was conducted at a different frequency (6.3 GHz) for comparison. In this case, no RF radiation was detected for either exposed or unexposed biofilm samples.

The interpretation of these observations as confirmation for the existence of RF radiation generated by *S. aureus* biofilms and that they demonstrate the biofilms actively respond to external RF signals is perplexing. Given the experimental situation, even inside an anechoic chamber, the frequency bands of 3.18 GHz and 3.45 GHz for the detected RF signals are in the range of the ubiquitous ambient cellular mobile communication spectrum. In contrast, the frequency of 6.3 GHz is well separated from the 3 GHz bands and is not a commonly found spectral component in the ever-present, over-the-air telecommunication domain. Moreover, the issue of electromagnetic compatibility and interference or spurious RF pick up of emissions from active instrumentation clocks and oscillator inside the RF anechoic chamber could also present potential complications.

## Discussion and conclusion

Public health concerns for the biological effects and safety of wireless RF radiation exposure are increasing with the rapid proliferation of cellular mobile telecommunication systems and devices. There is also lack of confidence about the efficacy of promulgated health safety limits, rules, and recommendations for wireless RF radiation including 5G used by these devices and systems. The currently promulgated RF exposure guidelines and standards apply predominantly to restrict short-term heating of RF radiation due to elevated tissue temperatures.

There are substantial incongruities and inconsistencies in the ICNIRP guidelines and IEEEICES standards. Furthermore, apart from the guideline’s irregularities, the biased assessments of the scientific database and less trustworthy appraisals such as many of the recent WHO sponsored systematic reviews make it difficult to reach a judgment with confidence. Some of the safety guidelines are irrelevant, debatable, and absent of scientific justification from the standpoint of safety and public health protection.

Full recognition of a public health risk takes time, and it is taking even longer these days given the fast pace of technological developments and rapidity at which they are launched into the commercial realm. The postulate of “An ounce of prevention is far better than a pound of cure” appears to have banished with little trace (39). Its mere mention under the current environment easily stirs robust rejoinders, with momentous opposition from those who may have profited from the massive marketing efforts. But given the growing ubiquity, is the premise of an “ounce of prevention” for RF radiation from cellphones and related wireless communication tools so far out of the question?

The question of how there can be such dissimilar assessments and inferences of the same scientific studies has persisted for some time. Less than strict enforcement of policies and procedures in research conduct or full disclosure of conflicts of financial and other interests can lead to failures in guiding and informing the development of transparent and consistent evaluations of scientific evidence for safety protection. Humans are not necessarily consistent or as reasonable as presumed. It is well known that politicians frequently make choices to promote self-interest or gain political advantage. To be fair, scientists can be driven by egocentric motives and are not immune to conflicts of interest. Indeed, science has never been devoid of politics—like it or not. Humans regularly make choices and judgments that challenge clear logic. Biases can impair rational thinking and lead to flawed decisions. “Groupthink can keep humans from being sensible and prevent the reaching of evidence-based conclusions” (30). Regrettably, groupthink or the herd mindset is as rife today as ever. “Has science become partisan? And the corollary, if science becomes partisan, is it science or politics, or would it be political science? Perhaps, science got wrapped up in politics and politics is intervening with science—a matter of guilelessly being politically correct of the willing” (82). When decisions are made through compromised judgment or not reached by cautiously weighing the scientific information they could lead to poor conclusions through biases.

Cellphones and wireless mobile communication technologies have enriched human lives. It is difficult to imagine contemporary lives without them. The deployment of 5G mobile technology is well underway with it heralded mm-wave performances. It is not evident whether the health effects of 5G mm-wave radiations would be analogous or not to previous generations of cellphone and wireless communication technologies, given the paucity of research on health effects of 5G mm-wave radiations. Without dispute, cellphones have provided direct benefits to multiple arenas of human endeavor that includes helping to safeguard our personal safety and security. Nonetheless, for the judgment on the health and safety of billions of users who are subjected to repeated, unnecessary levels of RF radiation over a protract length of time or even over their lifetimes, the verdict is still out. It is significant to note that cellphones have SAR ranging from 0.2 and 0.5 W/kg (83). Clearly, cellphones operate at a fraction of the SAR acceptable to IEEE-ICES and ICNIRP. It is conceivable that forthcoming developments would enable cellphone functions including data transmission at much lower exposure levels. Therefore, the ALARA—as low as reasonably achievable principle and practice —should be followed for RF health and safety when confronted with such divergent assessments of wireless RF radiation.

As noted, the recent announcement of termination of NIH-NTP’s RF effects research program on how RF microwave radiation causes cancer practically halted most, if not all, biological research of RF radiation supported by the civilian U.S. government. On the other hand, the RadioBio initiative seems to suggest a paradigm shift in the U.S. military’s standard of operation procedures, away from a conviction of nothing but thermal effect could be associated with RF and microwaves. The new initiatives appear to allow exploration (and perhaps exploitation) of low-level, nonthermal biological response to RF radiation. In this regard, the recent publications from some of the military research laboratories may serve as telltales of more to come. These results are putting a spotlight on an atypical event, a paradigm shift in which a scientific investigation from an U.S. military research laboratory reporting a cytogenetic response or more specifically, an epigenetic role in the cellular response to low-level RF exposure, potentially, with major influences on gene activities.
